# Anti-CHAC1 exosomes for nose-to-brain delivery of miR-760-3p in cerebral ischemia/reperfusion injury mice inhibiting neuron ferroptosis

**DOI:** 10.1186/s12951-023-01862-x

**Published:** 2023-03-27

**Authors:** Yong Wang, Huicong Niu, Luyu Li, Jing Han, Zhuohang Liu, Min Chu, Xianyi Sha, Jing Zhao

**Affiliations:** 1 Department of Neurology, Minhang Hospital, Fudan University, Floor 16th, # 170 Xinsong Road, Shanghai, 201199 China; 2 Department of Dermatology, Shanghai Ninth People’s Hospital, Shanghai Jiaotong University School of Medicine, Shanghai, China; 3 State Key Laboratory of Medical Neurobiology, Department of Integrative Medicine and Neurobiology, Brain Science Collaborative Innovation Center, School of Basic Medical Sciences, Institutes of Brain Science, Fudan Institutes of Integrative Medicine, Fudan University, Shanghai, China; 4 Key Laboratory of Smart Drug Delivery, Ministry of Education, School of Pharmacy, Fudan University, Shanghai, 201203 China; 5 The Institutes of Integrative Medicine, Fudan University, 120 Urumqi Middle Road, Shanghai, 200040 China

**Keywords:** Cerebral ischemia, Exosomes, Ferroptosis, Intranasal administration, CHAC1

## Abstract

Ferroptosis plays a critical role in ischemic stroke, and anti-ferroptosis strategies were regarded as potentially effective measures. Based on ferroptosis-related mechanisms, this study aims to design and prepare anti-ferroptosis exosomes from adipose-derived mesenchymal stem cells (ADSC-Exo) for treating ischemic brain injury via intranasal (IN) administration. According to the bioinformatic analysis, CHAC1 was a key gene in the progress of ferroptosis in ischemic stroke. miR-760-3p can inhibit the expression of CHAC1 and may be abundant in ADSC-Exo. Therefore, ADSC-Exo were successfully isolated and the immunofluorescence showed that they can be efficiently delivered to the brain via IN administration. Additionally, IN administration of ADSC-Exo can effectively improve the neurobehavior function of mice after I/R, and improve the ferroptosis-related outcomes. As the immunofluorescence showed the co-localization of NeuN with CHAC1 obviously, we further evaluated the systematic effect of ADSC-Exo in an oxygen–glucose deprivation (OGD) mouse neuroblastoma cell line N2a model. The results showed that miR-760-3p in ADSC‐Exo contributed to their function in inhibiting ferroptosis by targeting CHAC1 in neurons. Collectively, the present study successfully designed and prepared anti-CHAC1 ADSC-Exo and suggested a promising exosome-based strategy for anti-ferroptosis therapy in cerebral ischemia/reperfusion injury.

**Graphical abstract:**

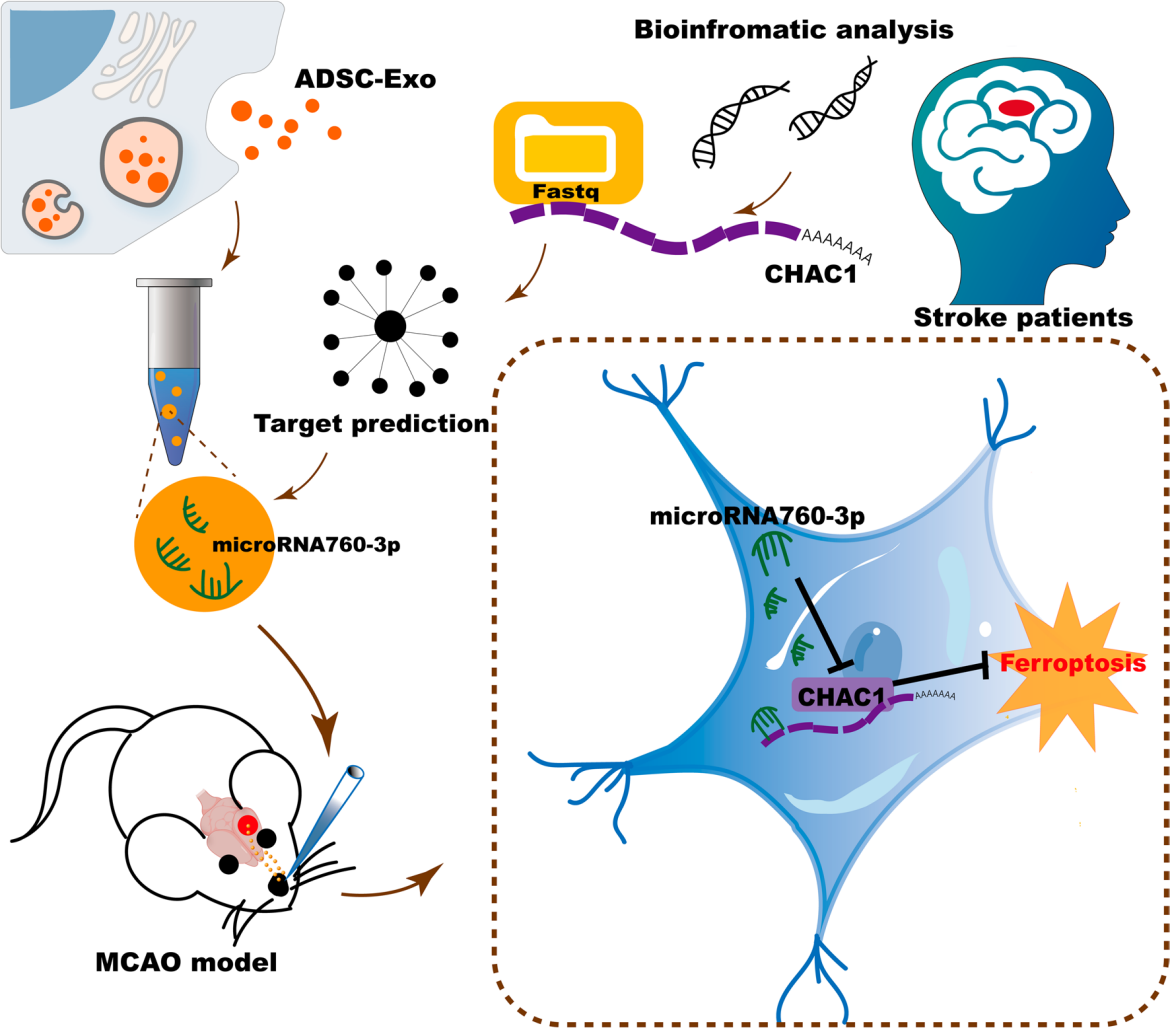

## Introduction

As the leading cause of long-term disability and the second leading cause of death worldwide, stroke remains one of the most prevalent and devastating diseases affecting the world population [1]. Approximately 70% of stroke cases are ischemic stroke characterized by occlusion of a major cerebral artery, usually the middle cerebral artery [2, 3]. Intravenous alteplase and/or mechanical thrombectomy are recommended effective treatments for acute ischemic stroke patients inducing reperfusion of the infarcted areas of the brain [4, 5]. However, brain damage typically continues to progress after reperfusion. This post-reperfusion damage is called ischemia–reperfusion (I/R) injury, which is triggered by the inflammatory response, reactive oxygen species (ROS), and excitotoxicity [6]. These pathological processes can further induce various kinds of cell death in the brain.

Ferroptosis, a newly identified type of cell death program, is characterized by the iron-dependent accumulation of lipid hydroperoxides to lethal levels [7, 8]. Ferroptosis has been identified and investigated in various neurological diseases including degenerative diseases, traumatic brain injury, hemorrhagic stroke, and ischemic stroke [7, 9]. A recent study showed that acyl-CoA synthetase long-chain family member 4 (ACSL4), an isozyme for polyunsaturated fatty acids (PUFAs) metabolism, was a novel regulator of ferroptosis and significantly influence the recovery of ischemic stroke [10]. Previous studies also showed that increased lipid peroxidation and decreased glutathione (GSH) levels were detected in ischemic stroke mice, and ferroptosis inhibitors such as Liproxstatin-1 and Ferrostatin-1 can promote the recovery of mice after ischemia/reperfusion injury [11, 12].

Although anti-ferroptosis therapy appears promising in ischemic stroke, these ferroptosis inhibitors only can be delivered into the brain by local stereotaxic injection or intravenous administration, which remains a barrier to its use. The local stereotaxic injection causes damage to the brain tissue because of the needle used for direct injection, while intravenous administration reduces the delivery efficiency into the brain across the brain–blood barrier [13]. Recently, intranasal (IN) administration is considered a reliable way of drug administration, given IN administration delivers therapeutic agents into the brain by way of the olfactory and trigeminal nerves, bypassing the BBB [13]. IN administration is more efficient than intravenous administration and less invasive than local stereotaxic injection [6]. Many previous studies have demonstrated the delivery efficiency of IN administration in delivering therapeutic agents to the brain and confirmed the positive effects of intranasal delivery in brain-related diseases [6, 14, 15]. Therefore, it is necessary to explore an anti-ferroptosis agent that can be delivered intranasally to the brain, which will be of great value to clinical applications.

As carriers released by cells, exosomes (Exo) have been shown to play an important role in the cross-talk between various cell types [16]. Exosomes contain a large number of biologically functional substances including proteins, lipids, DNA, and RNA [17]. They are capable of diffusing to neighboring cells or being transported to distant anatomic locations where the signals or information are transduced to specific recipient cells [18, 19]. Previous studies have demonstrated that IN administration of Exo is efficient for delivering Exo into neurons and microglia in the brain [6, 15]. Additionally, previous studies indicated that administration of Exo from adipose-derived mesenchymal stem cells (ADSC-Exo) not only achieves a comparable therapeutic effect of ADSCs but also effectively avoids the adverse effects of stem cell transplantation such as immunological rejection and tumorigenesis [20]. Previous studies have shown that ADSC-Exo can inhibit ferroptosis in acute liver injury [21] and hemorrhage stroke [22]. Although the effects of ADSC-Exo on ferroptosis in ischemic stroke have not been demonstrated, it is worth trying to explore this, especially delivery them by IN administration.

In the present study, the results of a bioinformatic analysis identified that CHAC1 was a key gene regulating ferroptosis in ischemic stroke, and the microRNAs (miRNAs) that target CHAC1 were predicted. miRNAs are non-coding short RNA molecules, which target the 3′-untranslated regions (3′-UTR) of specific mRNAs and regulate their translation. Various miRNAs have been reported to play a key role in several brain diseases by downregulating the expression of key proteins [23, 24]. MicroRNA-760-3p (miR-760-3p) is one of the several miRNAs targeting CHAC1, and it is abundant in ADSC-Exo derived from humans or mice confirmed by bioinformatic analysis. Then, ADSC-Exo were isolated and administered IN in a mouse middle cerebral artery occlusion (MCAO) model of ischemic stroke to evaluate their effects in inhibiting ferroptosis and promoting neurological recovery. Additionally, various assays were performed in an oxygen–glucose deprivation (OGD) cell model to further demonstrate the therapeutic effects of ADSC-Exo and its mechanism of anti-ferroptosis.

## Methods

### Ethics statement

Ten to twelve weeks old Male C57/BL6 were purchased from Beijing Vital River Laboratory Animal Technology Co., Ltd. (Beijing, China). The animal experiment protocol was approved by the ethical review board of Central Hospital of Minhang District, Shanghai, China, and was performed according to its guidelines strictly. Every effort was made to minimize the number of experimental animals and reduce animal suffering. From August 2022 to September 2022, 5 stroke patients were included according to the following criteria: (1) more than 18 years old; (2) with a final diagnosis of “cerebral infarction” confirmed by head CT or MRI; (3) first onset (4) onset time no more than 3 days; (5) informed consent was obtained from the patients or their legal representatives. Control subjects were 5 age and gender-matched healthy volunteers without a history of stroke or other vascular diseases (Tables 1, 2). Peripheral blood samples were collected through forearm veins from the participants, and Ethylene Diamine Tetraacetic Acid (EDTA) vacutainer blood collection tube was used to keep those samples. The clinical study was approved by the ethical review board of Central Hospital of Minhang District, Shanghai, China.

**Table 1:** Age and gender differences between patients and controls

	Stroke (n = 5)	Control (n = 5)	P value
Age, year, M ± SD	69 ± 11	73 ± 9	0.584
Sex, Male, N (%)	2 (40%)	2 (40%)	1.00

One-way ANOVA test and Pearson Chi-Square test were employed to examine the differences in age and sex between controls and patients as appropriate

*SD* standard deviation

**Table 2:** Clinical and radiological characteristics of stroke patients

Patients	Age	Sex	Diagnosis	TOAST classification	Infarct location
1	54	Male	Cerebral infarction	LAA	Local subacute cerebral infarction in medulla oblongata right side
2	69	Female	Cerebral infarction	LAA	Subacute left frontal lobe cerebral infarction
3	64	Female	Cerebral infarction	LAA	Subacute cerebral infarction at the left edge of brainstem
4	86	Female	Cerebral infarction	LAA	Small infarct in right temporal lobe
5	71	Male	Cerebral infarction	LAA	Subacute cerebral infarction in the right paraventricular body and bilateral centrum semiovale

*LAA* large artery atherosclerosis

### MCAO in mice

A total of 130 male C57/BL6 mice weighing 25–30 g received MCAO surgery, and 100 (76.9%) of them survived 3 days after the operation and were included in the follow-up experiment. Mice were anesthetized with 1% pentobarbital intraperitoneally and then received surgery using the modified Zea Longa method [25]. Briefly, a 6-0 nylon monofilament suture coated with silicon (Jialing Co., Ltd., Guangzhou, China) was inserted into the external carotid artery and then gently advanced into the internal carotid artery to block the middle cerebral artery. At 60 min post-occlusion, the filament was withdrawn and the reperfusion was allowed. For the mice assigned to the sham group, the filament was only inserted into the external carotid artery but did not block the middle cerebral artery. Techniques were used to improve the survival rate of mice including (1) Minimizing the destruction of muscles and connective tissue; (2) Mice need to wear eye masks because intense light stimulation from surgical lights causes severe dryness of the eyes; (3) Avoid bleeding and keep the surgical field clean and tidy; (4) Put the mice on the heating pad during the whole process under anesthesia, otherwise, they will easily die of hypothermia. Regional cerebral blood flow of MCAO animals was measured using Laser speckle imaging (Perimed, Sweden). Triphenyl tetrazolium chloride (TTC, Sigma-Aldrich, Saint Louis, MO, USA) staining was used to assess the infarct volume of MCAO mice 24 h after surgery.

### Identification of ferroptosis-related different expression genes (DEGs) and prediction of suitable anti-ferroptosis Exo based on the content of DEGs’ target miRNAs

Three mRNA expression datasets (GSE162955, GSE131712, and GSE58294) and 2 non-coding RNA datasets (GSE153752 and GSE79440) were downloaded from the Gene Expression Omnibus (GEO, https://www.ncbi.nlm.nih.gov/geo/) database by using the GEO query package of R software (version 4.1.1). GSE162955 contains the expression data of 6 brain samples from the infarct core of stroke patients and 6 from the healthy contralateral areas of them. GSE131712 contains the data of mouse ischemic cortex from 6 mice that received MCAO surgery and 6 control ones. GSE58294 included blood samples from 69 ischemic stroke patients and 23 control ones. GSE162955 was used to identify the ferroptosis-related DEGs, and the results were validated using GSE131712 and GSE58294. The DEGs of GSE162955 were identified by limma R package with |logFC|> 0.3 and P adj. < 0.05 and visualized by a Volcano plot using ggplot2. Then, 73 ferroptosis-related genes were downloaded from FerrDb (http://www.zhounan.org/ferrdb/). The up-regulated and down-regulated DEGs of GSE162955 were obtained to intersect with ferroptosis-promoted and ferroptosis-inhibited genes. The ferroptosis-related DEGs were identified and the visualized a Venn diagram. The target miRNAs of ferroptosis-related DEGs were predicted using starBase v2.0 [26] and the interaction network was visualized by Cytoscape software [27]. Subsequently, the miRNA datasets of exosomes from human ADSCs (GSE153752) and mouse ADSCs (GSE79440) were used to determine the target miRNAs of ferroptosis-related DEGs, which play a key role in inhibiting ferroptosis in ischemic stroke. The cross genes of miRNAs in exosomes and starBase-predicted miRNAs were selected as candidate target miRNAs. Additionally, the expression level of these candidate target miRNAs in mouse ADSC-Exo (GSE79440) was ranked, and the expression level of the highest expression target miRNA was assessed in GSE162955 and GSE131712.

### Isolation and characterization of ADSCs

ADSCs were isolated from 8-week-old male C57/BL6 mice. Mice were anesthetized with 1% pentobarbital intraperitoneally and then soaked in 70% alcohol for 10 min. Inguinal subcutaneous adipose tissue of mice was collected and cut into mince on ice quickly. Then the adipose tissue was incubated with 0.1% type I collagenase (Sigma-Aldrich, Saint Louis, MO, USA) at 37 °C for 45 min in a rotatory shaker at 110 rpm and shook the test tube violently by hand every 15 min. Low glucose Dulbecco’s modified Eagle medium (L-DMEM, Sigma-Aldrich, Saint Louis, MO, USA) containing 10% fetal bovine serum (FBS, Gibco, Carlsbad, CA) was used to stop the enzymatic reaction. After filtering through a 70 μm filter and centrifuging at 1200 rpm for 5 min, cells were resuspended by PBS buffer (Genom, Zhejiang, China). After centrifugation at 1200 rpm for 5 min again, cells were resuspended and seeded in a complete medium at 37 °C with a 5% concentration of carbon dioxide. The complete medium consisted of L-DMEM containing 10% FBS and 1% penicillin/streptomycin (Genom, Zhejiang, China), and was replaced every 2 days. Cells were harvested using 0.25% trypsin–EDTA (Genom, Zhejiang, China), and passage 2–5 cells were used to conduct experiments.

The surface markers of ADSCs were validated using the BD FACSVerse flow cytometer (BD Biosciences, USA). Briefly, cells were digested and resuspended with PBS buffer, and then incubated with the following antibodies at 4 °C for 30 min in dark: FITC anti-mouse CD45 (Cat# 103107, Biolegend), APC anti-mouse CD34 (Cat# 128611, Biolegend), PE anti-mouse CD105 (Cat# 120407, Biolegend), and PE/Cyanine anti-mouse/human CD44 (Cat# 103029, Biolegend). The multiple differentiation capabilities of ADSCs were assessed by adipose mesenchymal stem cell osteogenic differentiation medium (Cat# PD-025, Pricella, Wuhan, China) and adipogenic differentiation medium (Cat# PD-027, Pricella, Wuhan, China), and cells were stained by Oil red O staining solution (E607319, BBI, Shanghai, China) or alizarin red staining solution (A600144, BBI, Shanghai, China).

### Isolation, identification, and labeling of Exo

A complete medium containing exosome-free FBS was used to culture passage five ADSCs, and Exo were collected from ADSCs supernatant by ultracentrifugation according to a published protocol [28]. Briefly, ADSCs supernatant was collected and then subjected to centrifugation at 300*g*, 2000*g*, 10,000*g*, and 110,000*g*, and the resulting precipitate was resuspended with PBS buffer. The morphological characteristics of Exo were observed by transmission electron microscopy (TEM, Hitachi, Japan). A particle size analyzer (NanoFCM, Fujian, China) was used to analyze the size distribution of Exo. The protein markers of Exo were assessed by Western blot using the following antibodies: anti-CD63 (Abcam, Cambridge, UK) and tumor susceptibility gene 101(TSG101, Abcam, Cambridge, UK). PKH26 red fluorescent cell linker kit (Sigma-Aldrich, Saint Louis, MO, USA) was used to label the Exo according to the manufacturer’s instructions.

### Intranasal administration of ADSC-Exo

After MCAO surgery, mice were randomly divided into the PBS group and ADSC-EV group. Mice were placed in the supine position and anesthetized with isoflurane under spontaneous breathing conditions. For the mice in the ADSC-Exo group, 2 μl PBS containing 2 ug ADSC-Exo were administrated intranasally as drops with a micropipette tip every 2 min into alternating sides of the nasal cavity for a total of 10 min [29]. A total of 10 μg ADSC-Exo were delivered into the nasal cavity per day for 1–3 days after MCAO. The mice in the PBS group received some volume of PBS for 3 days using the same method.

### Neurobehavioral tests

The modified neurological severity score (mNSS), Rotarod test, Foot fault test, and Open-field test were used to evaluate the neurobehavioral function of mice. The mNSS was used to assess the neurological deficits of mice at 1d, 3d, 7d, 14d, and 28d after MCAO. The total scores of this scale ranged from 0 to a maximum of 14. Severity was graded as follows: 1–5 scores, mild; 6–10, moderate, 11–15, severe. The coordination function of the mice was assessed by recording the time that the mice fell from the rotarod. Before MCAO, mice were trained for 3 days on the rotarod and collected the baseline data before surgery. After surgery, the rotarod test was carried out at 3d, 7d, 14d, and 28d after MCAO with a speed of 5–40 rpm/min. The foot fault test was used to assess the grasping function of mice’s affected limb at 14d, and 28d after MCAO. Mice were required to go across a 1 m length horizontal ladder at a uniform speed and repeat 3 times. This procedure was recorded with a camera and the percentage of partial placement step numbers accounting for the total step numbers of the affected limb was calculated. The open-field test was used to evaluate the motor function of mice at 7d, 14d, and 28d after MCAO. Mice were placed in the center of a 50 × 50 cm plastic box and freely explored the area for 5 min. A video tracking system (ANYmaze, Stoelting, IL, USA) was used to capture the movement trajectories of the mice and calculate the total movement distance of the mice in the open field.

### Luciferase reporter assay

A luciferase reporter assay was performed to verify whether the miR-760-3p could bind to the 3’-UTR of the CHAC1 mRNA. Briefly, the wide-type mouse 3’-UTR sequences with the binding sites of miR-760-3p (Mouse_Chac1-WT) were cloned into PGL3-CMV luciferase reporter vectors (Genomeditech, Shanghai, China). The mutant mouse CHAC1 3’-UTR sequences (Mouse_Chac1-MUT) containing mutations at the miR-760-3p binding sites were cloned into PGL3-CMV luciferase reporter vectors. Then, N2a cells were co-transfected with the PGL3-CMV luciferase reporter vectors and either the miR-760-3p mimic or NC mimic for 48 h using Lipofectamine 3000 reagent (Invitrogen, Carlsbad, CA, USA). Finally, cells were collected and a dual luciferase reporter gene assay kit (Yeasen, Shanghai, China) was used to assess the luciferase activity.

### Western blot analysis

Protein samples were collected from mouse ischemic penumbra tissues or N2a cells. The concentrations of protein samples were assessed by a BCA protein assay kit (Epizyme, Shanghai, China). The primary antibodies used in this study are as follows: anti-CD63 (ab217345, 1:1000, Abcam), TSG101 (ab125011, 1:1000, Abcam), CHAC1 (15207-1-AP,1:1000, Proteintech), GPX4 (ab125066, 1:5000, Abcam), ACSL4 (sc-365230, 1:1000, Santa Cruz), and β-actin (81115-1-RR, 1:5000, Proteintech),

### Total RNA isolation and real-time quantitative polymerase chain reaction (RT-qPCR)

RNA was collected from serum samples of patients, mouse ischemic penumbra tissues, and N2a cells to verify the expression level of genes. Total RNA was extracted by Trizol reagent (Invitrogen, USA). RNA samples from total RNA were reverse-transcribed to cDNA using PrimeScriptTM RT reagent Kit (TAKARA, Japan), and RT-qPCR was performed on a Light Cycler thermal cycler system (Bio-Rad, USA) using SYBR^®^ Premix Ex Taq™ II (TAKARA, Japan). GPADH was used as the endogenous control of mRNA. The relative expression was normalized to that in the control group. The sequences of the primers used in the present study were shown in Table 3.

**Table 3:** Primers for mRNA real-time polymerase chain reaction

Gene		Primer
Human		
CHAC1	Forward	5′-GACGCTCCTTGAAGATCATGAG-3′
	Reverse	5′-CAGCAAGTATTCAAGGTTGTGG-3′
ACSL4	Forward	5′-TCATGCACCGTTTCCCTGAA-3′
	Reverse	5′-CATTTGCCGGAAAGCACACA-3′
hsa-miR-760	RT primer	5′-CTCAACTGGTGTCGTGGAGTCGGCAATTCAGTTGAGTCCCCA-3′
	Forward	5′-TCGGCAGGCGGCTCTGGGTCTG-3′
	Reverse	5′-CTCAACTGGTGTCGTGGA-3′
GPX4	Forward	5′-CCAAGTTTGGACACCGTCTCT-3′
	Reverse	5′-TCCTTCTCTATCACCAGGGGC-3′
GAPDH	Forward	5′-AATGGGCAGCCGTTAGGAAA-3′
	Reverse	5′-GCCCAATACGACCAAATCAGAG-3′
Mouse		
CHAC1	Forward	5′-AGTGTGGAAGCCGGACTTTG-3′
	Reverse	5′-CACTCGGCCAGGCATCTTGT-3′
mmu-miR-760-3p	RT primer	5′-CTCAACTGGTGTCGTGGAGTCGGCAATTCAGTTGAGTCCCCA CA-3′
	Forward	5′-TCGGCAGGCGGCTCTGGGTC-3′
	Reverse	5′-CTCAACTGGTGTCGTGGA-3′
GPX4	Forward	5′-TTCCTGGGCTTGTGTGCATC-3′
	Reverse	5′-TATCGGGCATGCAGATCGAC-3′
ACSL4	Forward	5′-AAAGACTGGCAGGAAGGTGGTTAT-3′
	Reverse	5′-GGACAATTCTTCAGTGCAGCTTCT-3′
GAPDH	Forward	5′-ATCATCAGCAATGCCTCCTG-3′
	Reverse	5′-ATGGACTGTGGTCATGAGTC-3′

### Immunofluorescence staining

The mice were anesthetized and their brains were removed after perfusion with 4% paraformaldehyde. After dehydration by sucrose, the brains were embedded with OCT compound (Sakura, Japan) and quick-frozen at -20℃, and then coronally cut into 30 μm thick sections. The brain sections were washed with PBST 3 times and incubated with CHAC1 antibody (15207-1-AP,1:200, Proteintech), IBA1 antibody (016-26721, 1:200, Wako), GFAP antibody (60190-1-Ig, 1:200, Proteintech), or NeuN antibody (66836-1-Ig, 1:200, Proteintech) overnight at 4 ℃. After washing with PBST 3 times, these brain sections were incubated with different secondary antibodies (Invitrogen, USA) for 1 h at room temperature. After washing the brain sections with PBST 3 times again, and then they were mounted with a DAPI-containing mounting medium (southembiotech, USA). Three sections from each mouse were observed and imaged by immunofluorescence microscopy (Keyence, Shanghai, China).

### Cell culture and OGD

The mouse neuroblastoma cell line N2a was obtained from Procell Life Science & Technology Co., Ltd. (Wuhan, China) and cultured in High glucose Dulbecco’s modified Eagle medium (H-DMEM, Sigma-Aldrich, Saint Louis, MO, USA) containing 10% FBS and 1% penicillin/streptomycin at 37 °C with 5% CO_2_. OGD treatment was used to simulate ischemia and hypoxia conditions in vitro. After replacing the complete medium with glucose-free DMEM, the N2a cells were cultured in a hypoxic chamber containing 95% N2 and 5% CO_2_ for 4 h. Then, the cells were cultured in the complete medium under normoxic culture conditions for 24 h. The N2a cells in the control group were not exposed to OGD. 10 μg/ml ADSC-Exo were administrated in the ADSC-Exo group.

### miRNA mimic and inhibitor transfection/CHAC1 overexpression

The miR-760-3p mimic, miR-760-3p inhibitor, NC mimic, and NC inhibitor were obtained from Genomeditech Co., Ltd. (Shanghai, China), and transfected by using Lipofectamine 3000 reagent (Invitrogen, Carlsbad, CA, USA). Overexpression of CHAC1 was achieved by integrating the CHAC1 genome (Genomeditech, Shanghai, China) into the N2a genome using lentivirus virus transfection with the multiplicity of infection (MOI) = 10.

### CCK8

The N2a cells were seeded in 96-well plates with 5 × 10^3^ cells per well, and cultured at 37 °C and 5% CO_2_ for 24 h. 24 h after receiving corresponding treatment, fresh complete medium containing 10% CCK8 solution (Beyotime, Shanghai, China) was added into each well and continued cultured for 2 h. Then, the absorbance value (OD value) of each well was assessed using a microplate reader at a wavelength of 450 nm.

### Lipid peroxidation malondialdehyde (MDA) assay

MDA assay kits (Beyotime, Shanghai, China) were used to assess the lipid peroxidation level in mice, N2a cells, and the serum of humans. The mice's brain tissue or N2a cells were lysed using RIPA lysis buffer (Beyotime, Shanghai, China), and then the supernatant was collected after centrifugation at 12,000*g* for 10 min. The supernatant was mixed with a working solution in a ratio of 2:1, and the mixture was heated at 100 °C for 15 min. After centrifugation at 1000*g* for 10 min, 200 μl supernatant of the mixture was added to 96-well plates and the OD value was assessed using a microplate reader at a wavelength of 532 nm. The concentration of MDA was calculated according to the content of MDA and protein in each sample. As for the serum sample of humans, the concentration of MDA can be directly calculated based on the standard curve.

### Flow cytometry with the C11-BODIPY probe

Lipid peroxidation in cells was assessed with the live cell analysis reagent C11-BODIPY 581/591 (Thermo Fisher Scientific, MA, USA). The N2a cells were seeded at 2 × 10^5^ cells/well into 6-well plates and cultured at 37 °C and 5% CO_2_ for 24 h. After receiving different treatments, cells were washed with PBS buffer 2 times and incubated with C11-BODIPY (10 μM) staining solution in a culture medium in the dark for 30 min at 37 °C. Then, the cells were harvested with 0.25% trypsin solution and washed with PBS buffer 2 times. After being resuspended in PBS buffer, the cells were immediately analyzed with a flow cytometer.

### Iron assay kit

The determination of intracellular ferrous iron level (Fe^2+^) uses the iron assay kit (ab83366, Abcam). Firstly, samples were collected, washed with cold PBS, and homogenized in iron assay buffer, then collected supernatant and incubated. Finally, the iron probe was added to each well, mixed, and incubated at 37 °C for 60 min protected from light, and the content was immediately measured on a colorimetric microplate reader (OD 593 nm).

### FerroOrange probe

Intracellular ferrous iron level (Fe^2+^) of N2a cells was detected by FerroOrange Probe (F374, Dojindo). The N2a cells were seeded at 1 × 10^4^ cells/well into 96-well plates and cultured at 37 °C and 5% CO_2_ for 24 h. After receiving different treatments, cells were washed with PBS buffer 2 times and incubated with FerroOrange (1 μM) staining solution in the dark for 30 min at 37 °C. then the cells were immediately analyzed with a colorimetric microplate reader (Ex: 543 nm, Em: 580 nm).

### GSH assay

The Glutathione Assay Kit (S0053, Beyutime) was used for these experiments. First, collected and prepared cellular samples according to the manufacturer’s instructions. Then add the sample to the 96-well plate. 150 μL total glutathione detection working solution was added, and incubate at room temperature for 5 min. Finally, 50 μL 0.5 mg/ml NADPH solution was added, mixed, and incubated for 25 min, and measured with a colorimetric microplate reader (A412).

### Statistical analysis

SPSS 23.0 software (IBM Corporation, NY, USA) and GraphPad Prism 9.0 (GraphPad Software Inc., CA, USA) were used for data analyses in this study. Continuous data are presented as mean ± standard deviation (SD) or median (interquartile range) based on the normality and homogeneity of variance. For normally distributed data, significant differences between the 2 groups were analyzed using Student’s t-test. Differences among multiple groups were analyzed by one-way analysis of variance (ANOVA) test. For data not at normal distribution, the Mann–Whitney U test or Kruskal–Wallis test was applied. A value of p < 0.05 was considered significant.

## Results

### CHAC1 was identified as the ferroptosis-related DEG in ischemic stroke and prediction of ADSC-Exo as a suitable anti-ferroptosis agent

**Fig. 1:**
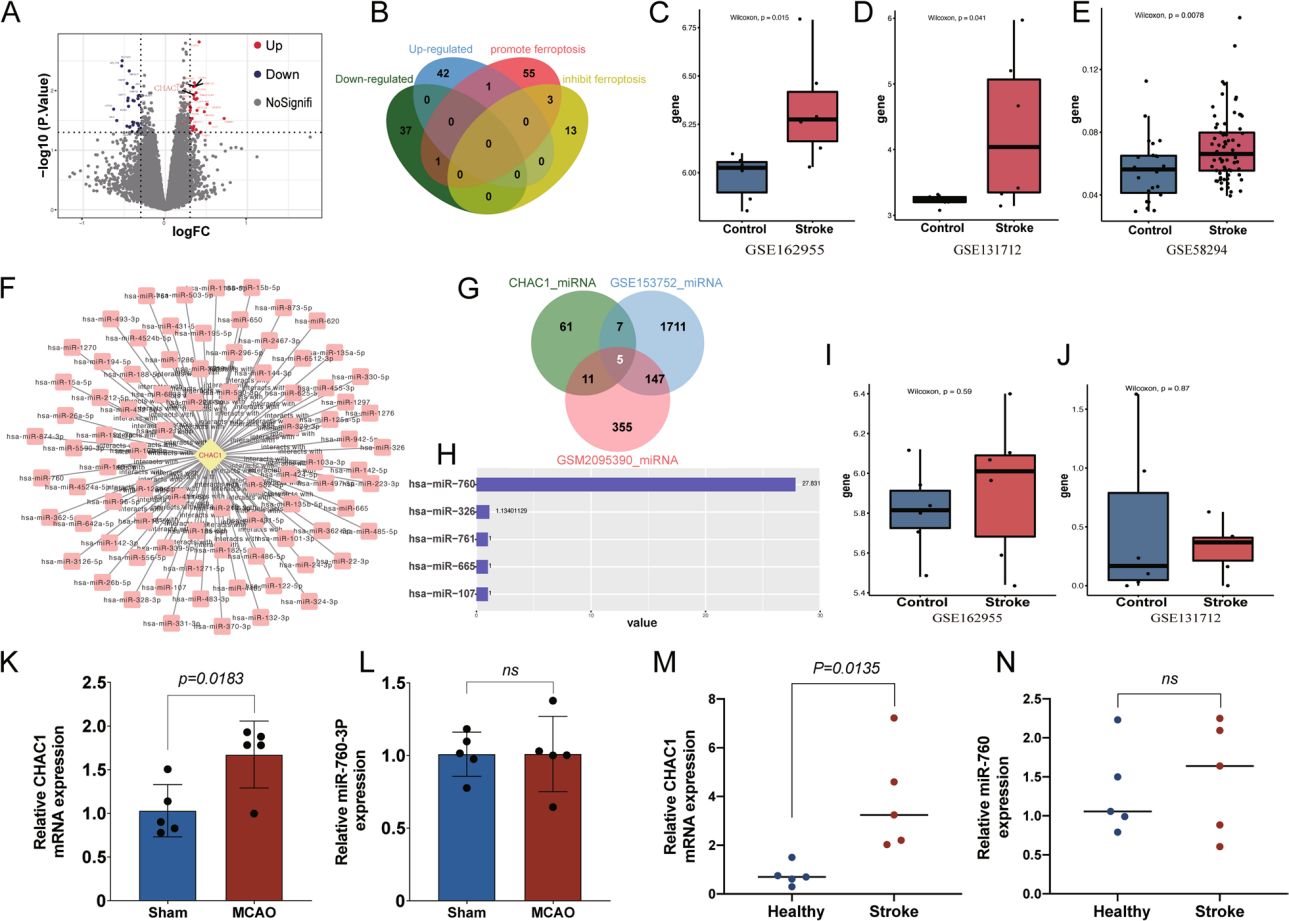
Identification of ferroptosis-related Different Expression Genes (DEGs) in ischemic stroke and prediction of ADSC-Exo as a suitable anti-ferroptosis agent based on the content of DEGs’ target miRNAs. **A** The volcano plot revealed 43 upregulated genes and 38 down‐regulated genes in the infarct core compared to healthy contralateral areas. **B** The ferroptosis-related DEGs were identified by a Venn diagram. **C** The CHAC1 expression in infarct core and healthy contralateral areas from a human dataset. **D** The CHAC1 expression of brain tissues at MCAO and control groups from a mouse dataset. **E** The CHAC1 expression of serum at the ischemic stroke and healthy groups from a human dataset. **F** The target miRNAs of CHAC1 predicted by starBase v2.0. **G** The cross miRNAs in ADSC-Exo and starBase-predicted miRNAs were identified by a Venn diagram. **H** The candidate target miRNAs in human ADSC-Exo were ranked. **I** The miR-760 expression in infarct core and healthy contralateral areas from a human dataset. **J** The miR-760-3p expression of brain tissues at MCAO and control groups from a mouse dataset. **K** The CHAC1 expression of brain tissues at MCAO and sham groups in mice, *n* = 5 **L** The miR-760-3p expression of brain tissues at MCAO and sham groups in mice, *n* = 5 **M**. The CHAC1 expression of serum from ischemic stroke and healthy people, *n* = 5. **N** The miR-760 expression of serum from ischemic stroke and healthy people, *n* = 5

To investigate the specific genes promoting ferroptosis in ischemic stroke, a comprehensive bioinformatic analysis was conducted. A total of 81 DEGs were identified between the infarct core and healthy contralateral areas of stroke patients, and 43 DEGs were up-regulated in the infarct core while 38 were down-regulated (Fig. 1A). The 43 up-regulated and 38 down-regulated DEGs were obtained to intersect with 60 ferroptosis-promoted and 16 ferroptosis-inhibited genes, and 1 ferroptosis-promoted DEG, CHAC1, was identified finally (Fig. 1B). The high expression of CHAC1 was further validated in datasets from stroke patient’s infarct core (*p* < 0.05, Fig. 1C), mouse ischemic cortex (*p* < 0.05, Fig. 1D), and blood samples of patients (*p* < 0.01, Fig. 1E). Then, the target miRNAs of CHAC1 were predicted using starBase v2.0 (Fig. 1F), and the candidate target miRNAs included in ADSC-Exo were selected as the 5 cross miRNAs in exosomes and starBase-predicted miRNAs (Fig. 1G). These 5 candidate target miRNAs include miR-760, miR-326, miR-761, miR-665, and miR-107, and the miR-760-3p was the most abundant in ADSC-Exo (Fig. 1H). The expression of miR-760 was further validated in datasets from infarct core tissue and blood samples of ischemic stroke patients. The results showed that the expression level of miR-760 in the stroke group was similar to that in the control group (*p* > 0.05, Fig. 1I, J), which indicated that miR-760 was important and shortage in patients after ischemic stroke, and it is necessary to supplement the miR-760 with ADSC-Exo. As miR-760-3p was the cross-mature sequence of miR-760 among humans and mice ADSC-Exo, miR-760-3p was selected in the following further research. Subsequently, the expression of CHAC1 and miR-760-3p were confirmed with RT-qPCR in ischemic stroke patients and mice, and the results were consistent with the bioinformatic analysis (Fig. 1K–N).

### Isolation and identification of ADSC-Exo, and investigation of the delivery efficiency of ADSC-Exo to MCAO mice brain via IN administration

**Fig. 2:**
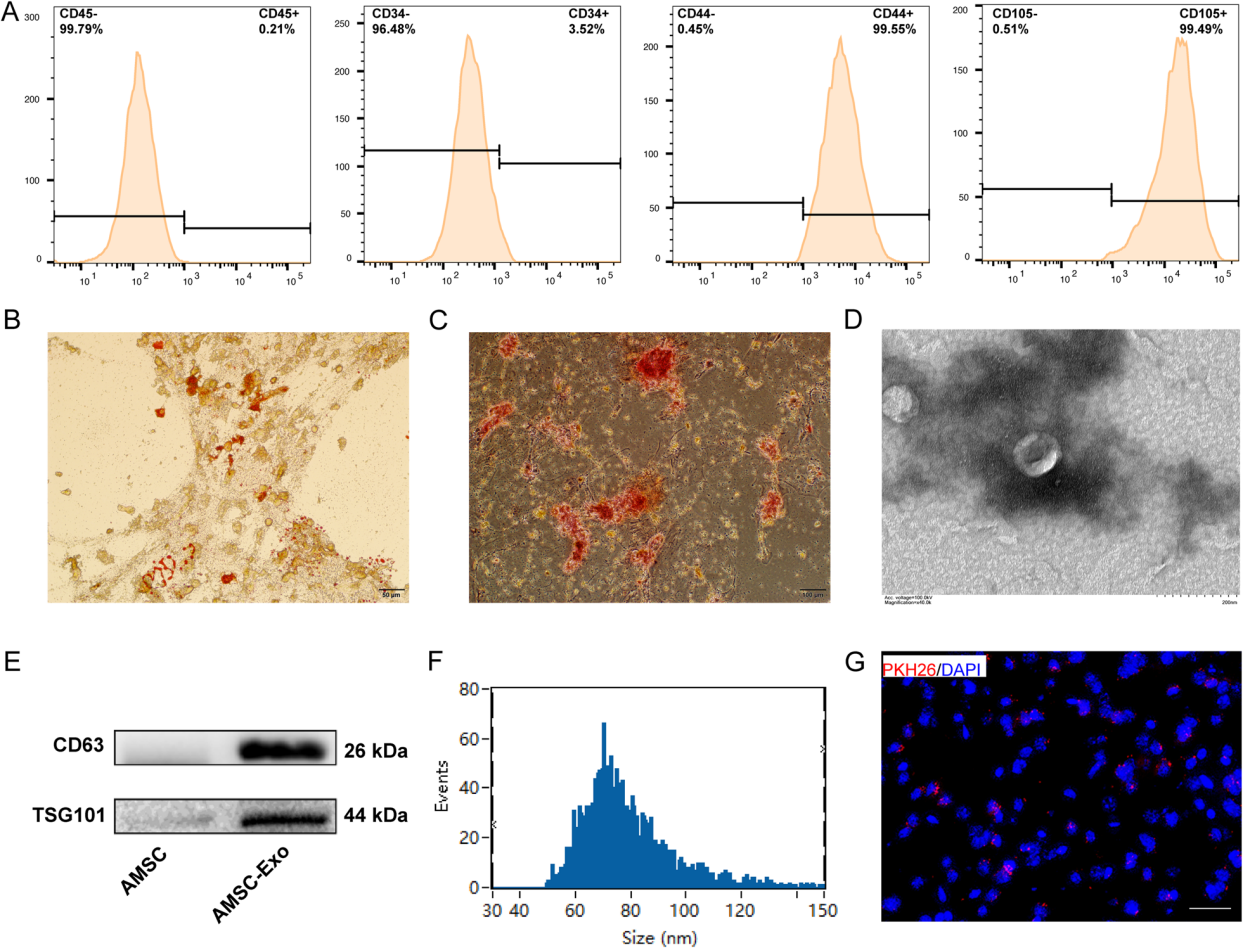
Characterization of ADSCs and ADSC-Exo. **A** Flow cytometric analysis of ADSCs surface markers CD44 and CD105, and negative markers CD45 and CD34. **B** Oil Red O staining showed a large number of red oil droplets in the cytoplasm (white arrows). Scale bars = 50 μm **C**. Alizarin red staining showed the red calcium nodules (white arrows) formed in cells. Scale bars = 100 μm **D** Representative TEM image of ADSC-Exo (white arrows). Scale bars = 200 nm. **E** Expressions of the ADSC-Exo markers CD63 and TSG101 were confirmed by Western blot. **F** Size distribution of ADSC-Exo measured by NanoFCM. **G** PKH‐26 labeled ADSC-Exo (Red) were detected around the nucleus (Blue) in the ipsilateral hemisphere of MCAO mice brain. Scale bars = 20 μm

ADSCs were isolated from C57/BL6 mice, and the expression of each cluster of differentiation (CD) marker in the second passage (P2) of them was detected by flow cytometry. As shown in Fig. 2A, CD45, and CD34 were negative expressions, while CD44 and CD105 were positive expressions, which meet the criteria for identifying ADSCs [30]. Oil Red O staining and Alizarin Red staining confirmed the adipogenic and osteogenic differentiation of ADSCs, respectively (Fig. 2B, C). ADSC-Exo were extracted from the medium of P2-5 primary ADSCs. As shown in Fig. 2D, A typical cup-shaped morphology was confirmed by TEM. Western blot was used to assess the protein markers expressed in exosomes, and the results revealed the abundance of CD68 and TSG 101 (Fig. 2E). Particle size analysis revealed that the mean size of ADSC-Exo was 78.88 ± 16.12 nm (Fig. 2F). All these results indicated that ADSC-Exo were successfully isolated. Additionally, PKH‐26 labeled ADSC-Exo (Red) were detected around the nucleus (Blue) in the ipsilateral hemisphere of MCAO mice brain, which indicated that the delivery efficiency of ADSC-Exo via IN administration was high (Fig. 2G).

### Ferroptosis occurred in ischemic stroke mice and patients

**Fig. 3:**
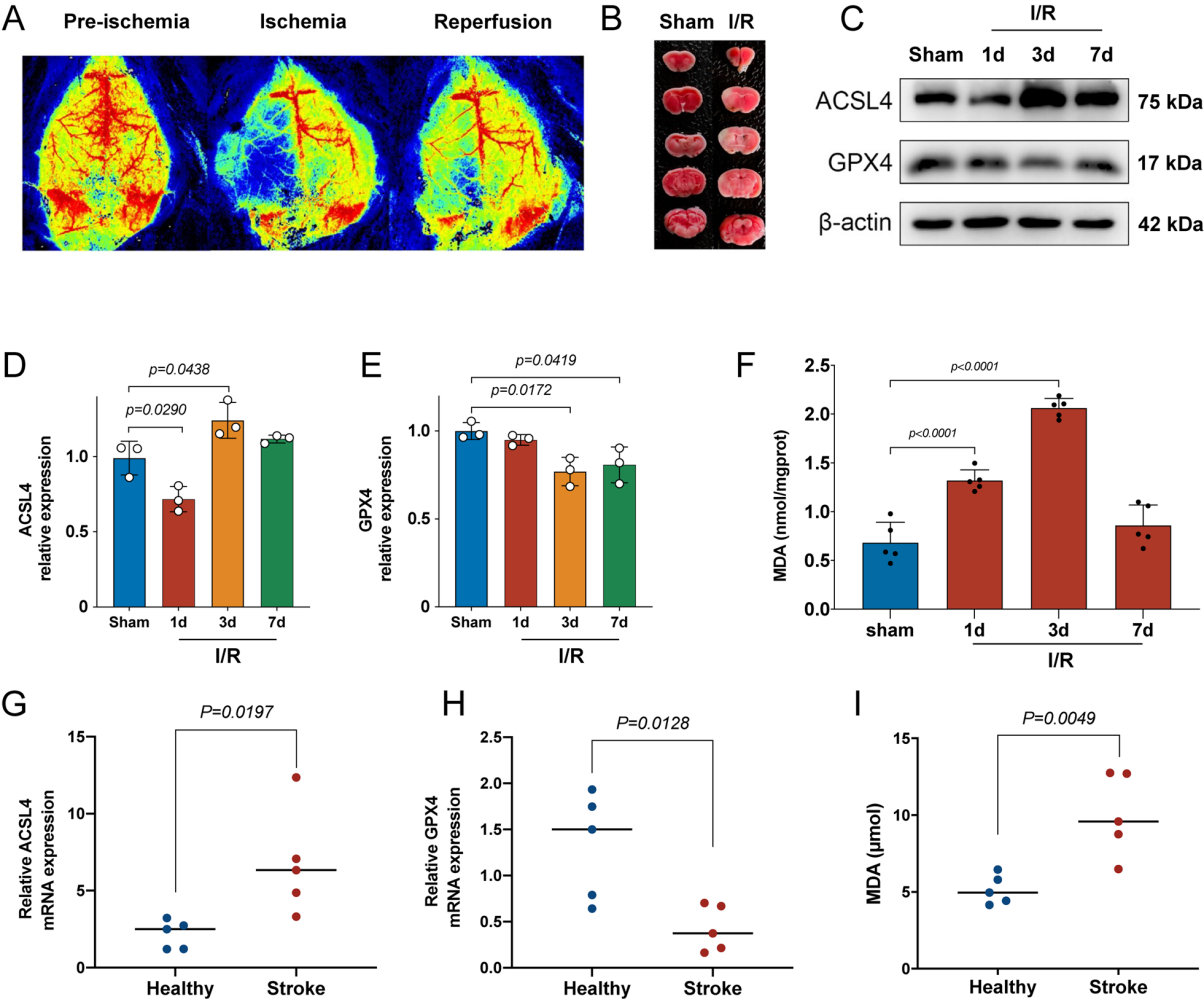
Ferroptosis occurred in ischemic stroke mice and patients. **A** Representative photomicrographs of laser speckle imaging for ischemic stroke mice. **B** Representative photomicrographs of brain coronal sections stained with triphenyl tetrazolium chloride (TTC) staining for ischemic mice at 24 h after MCAO. **C** Western blot analysis of ferroptosis markers ACSL4 and GPX4 at different time points after MCAO in mice, *n* = 3. **D** Quantification of the protein expression of ACSL4, *n* = 3. **E** Quantification of the protein expression of GPX4, *n* = 3. **F** Quantification of the malondialdehyde (MDA) at different time points after MCAO in mice, *n* = 5. **G**, **H** The mRNA expression levels of ACSL4 and GPX4 were detected by RT-qPCR in the serum from healthy volunteers and ischemic stroke patients, *n* = 5. **I** The amount of MDA detected in the serum from healthy volunteers and ischemic stroke patients, *n* = 5

To investigate whether ferroptosis occur or not in ischemic stroke mice and patients, the expression level of ferroptosis-related protein markers and lipid peroxidation MDA were assessed in MCAO mice and ischemic stroke patients. As shown in Fig. 3A, the laser speckle imaging indicated that the regional cerebral blood was blocked during ischemia and slight recovery after reperfusion. The results of TTC staining showed that the infarct volume of the brain was obvious 24 h after MCAO (Fig. 3B). These results indicated that the MCAO mice model was successfully established in this study. After MCAO, ACSL4 was up-regulated while GPX4 was down-regulated. The protein expression level of ACSL4 in mice at 3d after MCAO was the highest, and GPX4 in mice at 3d after MCAO was the lowest (Fig. 3C–E). The concentration of MDA in mice at 3d after MCAO was also the highest (Fig. 3F). These results indicated that ferroptosis occurred in mice after MCAO, and the condition may be most serious at 3d after MCAO. Additionally, the amount of MDA and the mRNA expression level of ACSL4 and GPX4 were significantly different between ischemic stroke patients and healthy participants (Fig. 3G–I), which was consistent with these results identified in mice.

### Intranasal administration of ADSC-Exo improved neurobehavior function and inhibit ferroptosis after ischemic stroke in mice

**Fig. 4:**
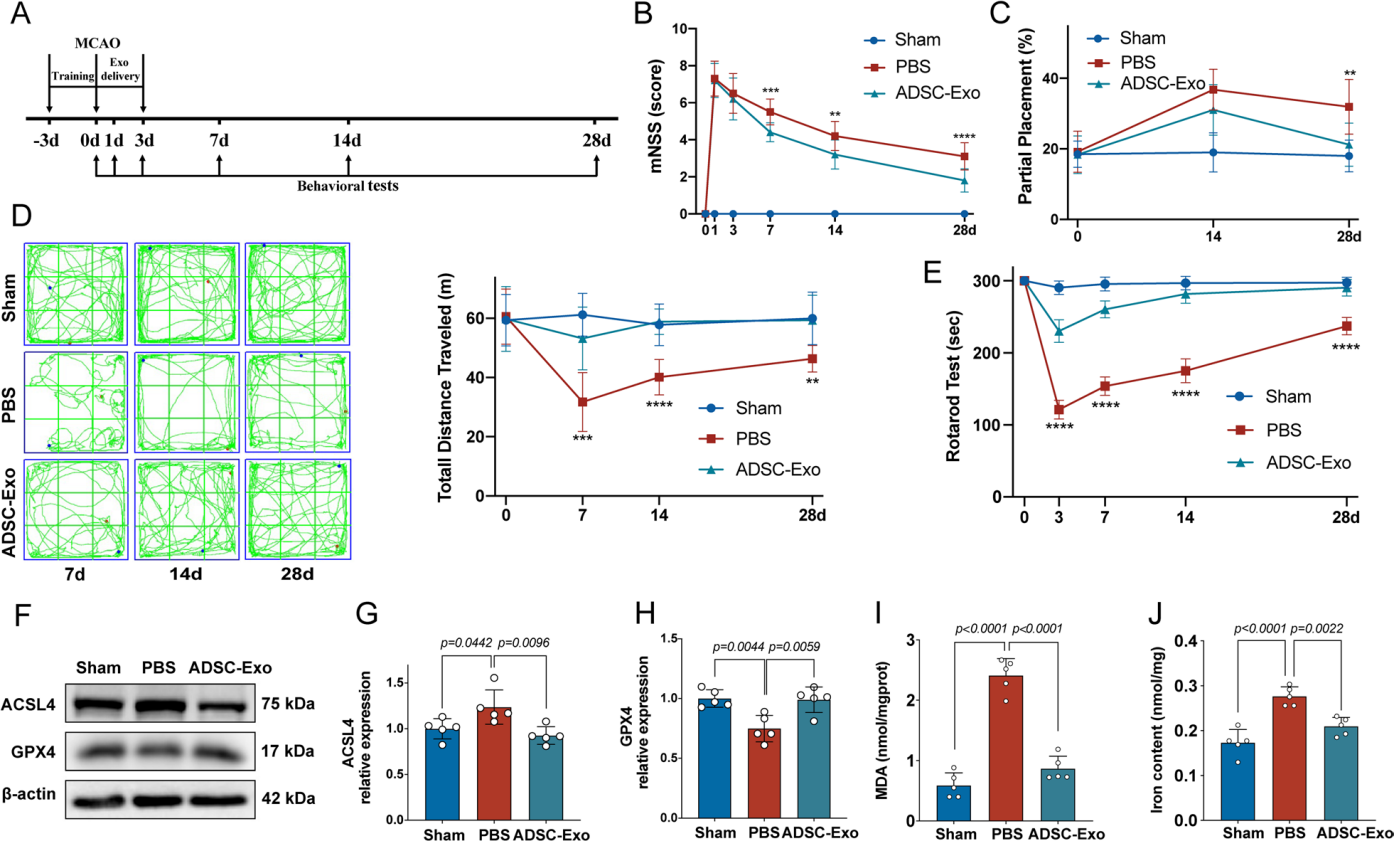
Intranasal administration of ADSC-Exo improved neurobehavior function and inhibit ferroptosis after ischemic stroke in mice. **A** Experimental schedule. ADSC-Exo intranasal administration was performed daily for 1–3 days after MCAO. **B** The neurological deficits were assessed by mNSS evaluation before MCAO and 1, 3, 7, 14, and 28 days after the operation, *n* = 10. **C** The grasping function of mice’s affected limbs before MCAO and 14 and 28 days after the operation was assessed by foot fault test,* n* = 10. **D** The motor function before MCAO and 7, 14, and 28 days after the operation was assessed by open-field test, *n* = 10. **E** The coordination function before MCAO and 3, 7, 14, and 28 days after the operation was assessed by the rotarod test, *n* = 10. **F** Ferroptosis-related protein markers ACSL4 and GPX4 expression detected by Western blot, *n* = 5. **G** Quantification of the protein expression of ACSL4, *n* = 5. **H** Quantification of the protein expression of GPX4, *n* = 5. **I** Quantification of the malondialdehyde (MDA), *n* = 5. **J** Quantification of ferrous iron level (Fe^2+^), *n* = 5. **p < 0.01, ***p < 0.001, ****p < 0.0001

To investigate the effects of ADSC-Exo on ischemic stroke mice, an experimental schedule was designed as shown in Fig. 4A. The mNSS showed that the neurological deficits of mice in the ADSC-Exo group were significantly less than that in the PBS group at 7d, 14d, and 28d after MCAO (p < 0.01, Fig. 4B). The rotarod test showed that the mice in ADSC-Exo group can stay on the rotarod longer than that in PBS group at 3d, 7d, 14d, and 28d after MCAO (p < 0.0001, Fig. 4C). The foot fault test showed that the grasping function of mice’s affected limb in ADSC-Exo group was better than that in PBS group at 28d (p < 0.01, Fig. 4D). In the open-field test, the total movement distance of mice in ADSC-Exo group was longer than that in PBS group at 7d, 14d, and 28d after MCAO (p < 0.01, Fig. 4E). These results indicated that ADSC-Exo can effectively improve the neurobehavior function of I/R mice. To verify the effects of ADSC-Exo on inhibiting ferroptosis in mice after MCAO, the protein markers and MDA were assessed. The protein expression of ACSL4 in mice of the ADSC-Exo group was significantly lower than that in the PBS group at 3d after MCAO (*p* < 0.01, Fig. 4F, G), while GPX4 in mice of the ADSC-Exo group was significantly higher (*p* < 0.01, Fig. 4F, H). In the lipid peroxidation MDA assay, the concentration of MDA in mice of the ADSC-Exo group was lower than that in the PBS group at 3d after MCAO (*p* < 0.0001, Fig. 4I). As for ferrous iron level (Fe^2+^), the amount of Fe^2+^ in mice of ADSC-Exo group was lower than that in PBS group at 3d after MCAO (*p* < 0.01, Fig. 4J). These results indicated that ADSC-Exo can inhibit ferroptosis in ischemic stroke mice.

### CHAC1 was up-regulated in mouse neurons at 3d after MCAO and ADSC-Exo were taken up by neurons in vivo and in vitro

**Fig. 5:**
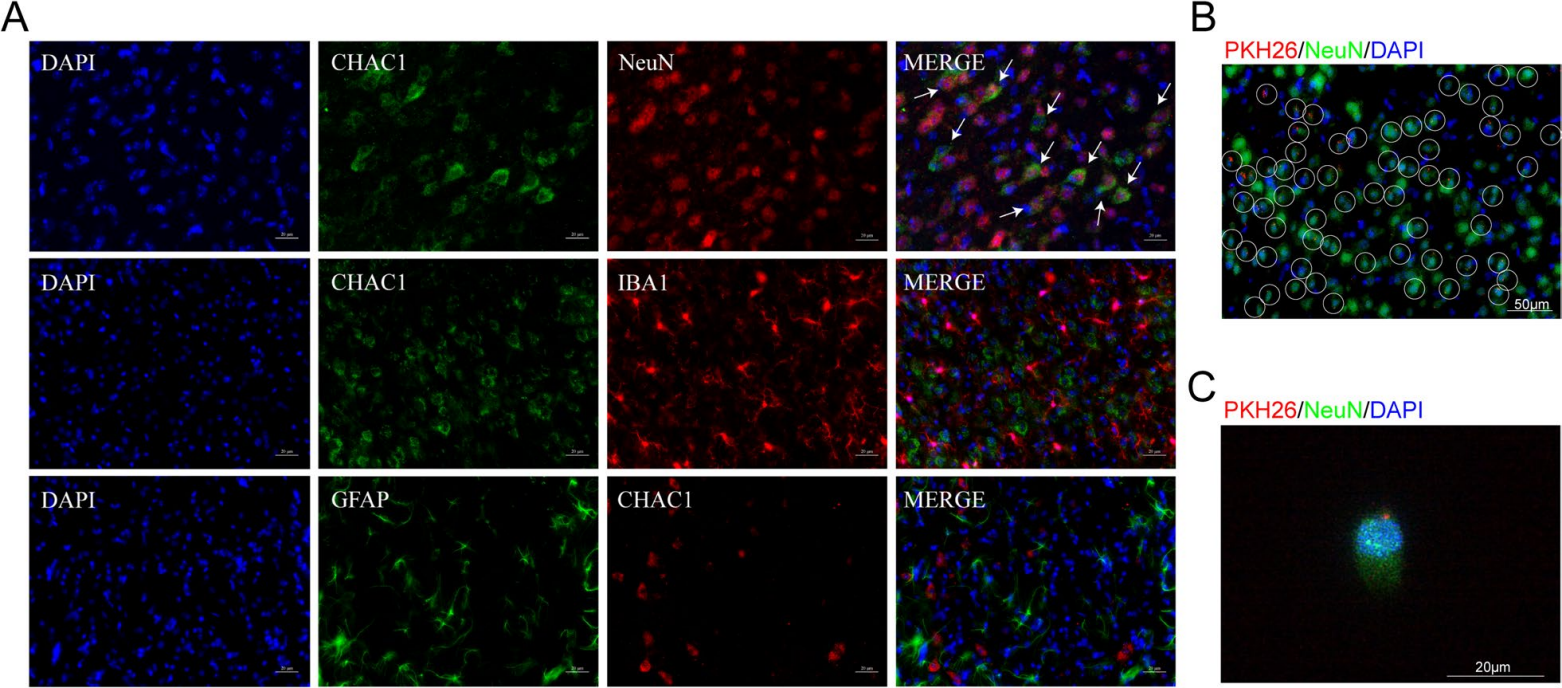
CHAC1 was up-regulated in mouse neurons at 3d after MCAO and ADSC-Exo were taken up by neurons in vivo and in vitro*.* **A** The immunofluorescence showed the co-localization of NeuN with CHAC1 obviously, while no co-localization of CHAC1 with IBA1 or GFAP was identified. White arrows indicated the co-localization of NeuN with CHAC1. Scale bars = 20 μm. **B** Immunofluorescence imaging showed the uptake of ADSC-Exo by neurons in vivo. PKH26 labeled ADSC-Exo (Red) were intranasally administered to the mouse at 24 h after MCAO. The internalization of ADSC-Exo by NeuN + neuron (Green) was detected 6 h after the administration. The white circle indicated ADSC-Exo internalized by neuron. Scale bar = 50 μm. **C** PKH‐26 labeled ADSC-Exo (Red) were taken up by NeuN + N2a cells (Green). PKH‐26 labeled ADSC-Exo were incubated with N2a cells for 6 h. Scale bar = 20 μm

To investigate the target cells on which CHAC1 mainly takes effect, we performed CHAC1/NeuN, CHAC1/IBA1, and CHAC1/GFAP double-staining. As shown in Fig. 5A, the immunofluorescence showed the co-localization of NeuN with CHAC1 obviously, while no co-localization of CHAC1 with IBA1 or GFAP was identified. These results indicated that CHAC1 mainly promote ferroptosis in mouse neuron at 3d after MCAO. Subsequently, we examined the uptake of ADSC-Exo by neurons in mice after MCAO. After 6 h of intranasal administration, immunofluorescence images showed the localization of PKH26-labeled ADSC-Exo in the cytoplasm of NeuN^+^ neuron (Fig. 5B). We also examined the uptake of ADSC-Exo by N2a cells. After 6 h of incubation with N2a cells, PKH26-labeled ADSC-Exo were detected in the cytoplasm of N2a cells (Fig. 5C).

### CHAC1 was verified as a downstream target gene of miR-760-3p in N2a cells

**Fig. 6:**
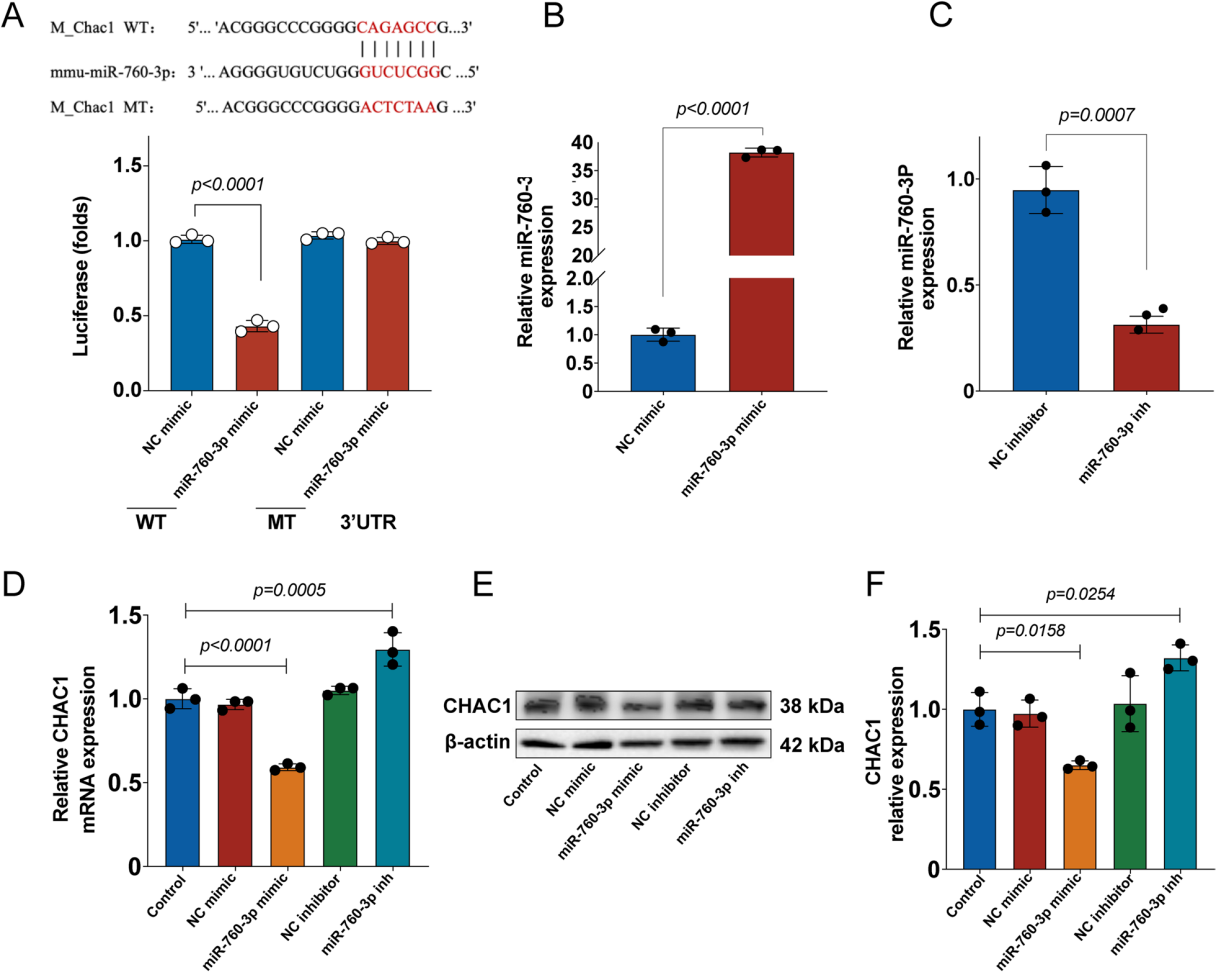
CHAC1 was verified as a downstream target gene of miR-760-3p in N2a cells. **A** A luciferase reporter assay verified the miR-760-3p could bind to the 3′-UTR of the CHAC1 mRNA, *n* = 3. **(B)** The miR-760-3p mimic elevated miR-760-3p levels in N2a cells by RT-qPCR, *n* = 3. **(C)** The miR-760-3p inhibitor suppressed miR-760-3p levels in N2a cells by RT-qPCR, *n* = 3. **(D)** The CHAC1 mRNA expression was assessed by RT-qPCR, *n* = 3. **(E, F)** The CHAC1 protein expression was assessed by Western blot, *n* = 3

Previous bioinformatic analysis indicated that CHAC1 was the downstream target gene of miR-760-3p. To verify whether the miR-760-3p could bind to the 3′-UTR of the CHAC1 mRNA, a luciferase reporter assay was performed. The luciferase reporter assay showed that the miR-760-3p mimic inhibited the luciferase activity of the CHAC1-WT seed compared with the NC mimic. However, the miR-760-3p mimic showed no effect on the luciferase activity of the CHAC1-MT seed (Fig. 6A). Subsequently, the effect of the miR-760-3p mimic and miR-760-3p inhibitor were assessed in N2a cells. The results of RT-qPCR showed that the miR-760-3p mimic elevated miR-760-3p levels, while the miR-760-3p inhibitor suppressed miR-760-3p levels (Fig. 6B, C), which suggested that miR-760-3p was successfully over-expressed and knocked down in N2a cells. Moreover, RT-qPCR and western blot analysis showed that miR-760-3p mimic reduces the expression of CHAC1, while miR-760-3p inhibitor increases the expression of CHAC1 (Fig. 6D–F). All these results indicated that chac1 was the downstream target gene of miR-760-3p in N2a cells.

### miR-760-3p inhibitor reversed the effects of ADSC-Exo on CHAC1 protein downregulation and ferroptosis inhibition

**Fig. 7:**
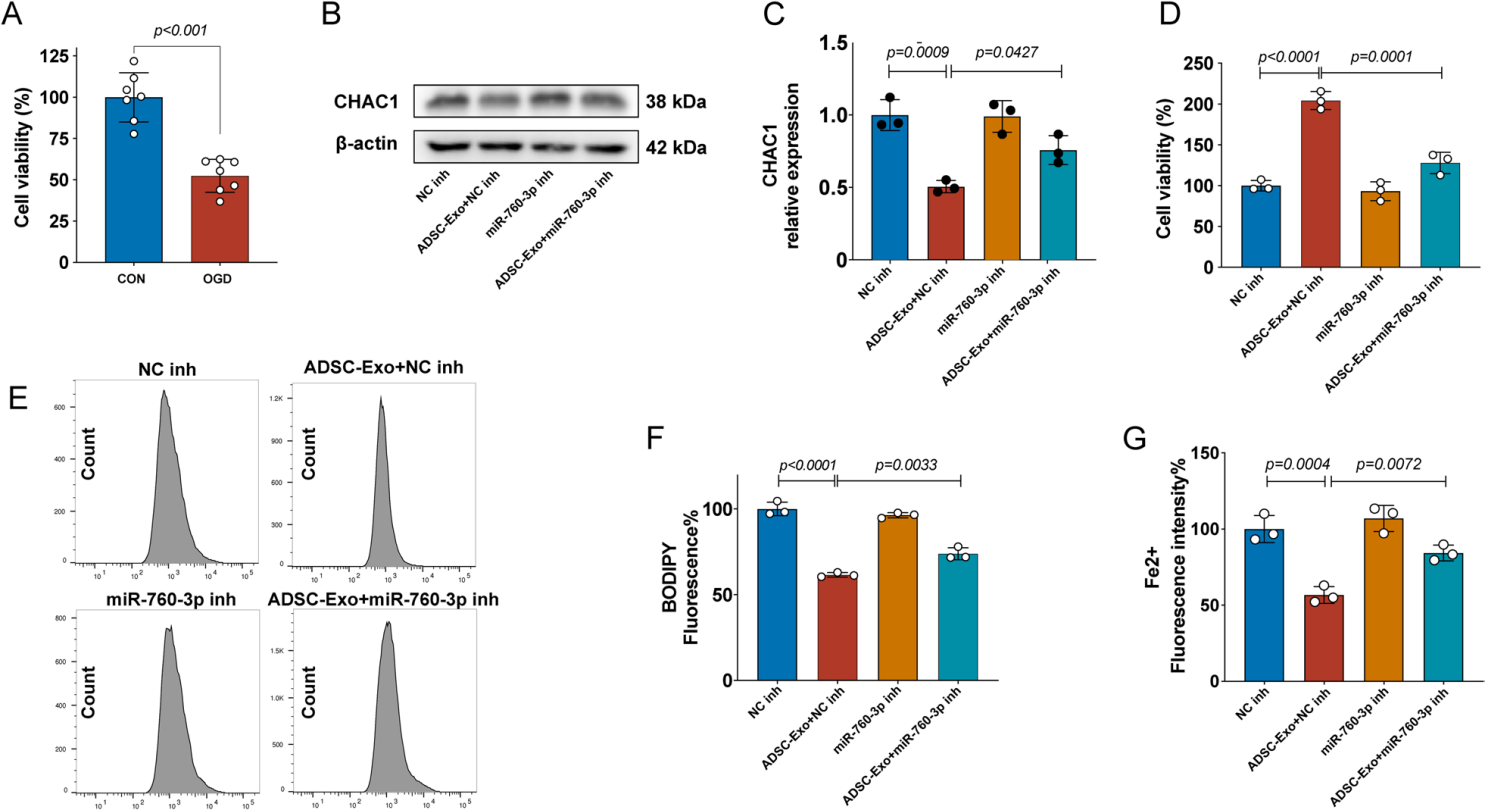
miR-760-3p inhibitor reversed the effects of ADSC-Exo on CHAC1 protein downregulation and ferroptosis inhibition. **A** Successfully established of OGD model of N2a cells confirmed by CCK-8 assay, *n* = 7. **B**, **C** The CHAC1 protein expression of OGD-treated N2a cells in different groups was assessed by Western blot, *n* = 3. **D** The survival rate of OGD-treated N2a cells in different groups was assessed by CCK-8 assay, *n* = 3. **E, F** The lipid peroxidation of OGD-treated N2a cells in different groups was assessed by the C11-BODIPY probe**,** *n* = 3. **G** The ferrous iron level (Fe^2+^) of OGD-treated N2a cells in different groups was assessed by the FerroOrange probe**,** *n* = 3

To investigate whether ADSC-Exo can downregulate the expression of CHAC1 and inhibit ferroptosis and whether these effects of ADSC-Exo are associated with miR-760-3p, we assessed the CHAC1 levels and ferroptosis-related outcomes in OGD-treated N2a cells. The CCK-8 assay confirmed that the OGD model of N2a cells was successfully established in this study (Fig. 7A). The western blot analysis showed that ADSC-Exo significantly reduced the CHAC1 expression, while miR-760-3p inhibitor reversed this effect of ADSC-Exo (Fig. 7B, C), suggesting that ADSC-Exo downregulate the expression of CHAC1 through miR-760-3p. The CCK-8 assay, FerroOrange probe, and C11-BODIPY probe showed that ADSC-Exo increased the survival rate (Fig. 7D) and reduced the lipid peroxidation (Fig. 7E, F) and ferrous iron levels (Fig. 7G) of OGD-treated N2a cells, while these effects of ADSC-Exo were reversed by miR-760-3p inhibitor, indicating that ADSC-Exo inhibit ferroptosis in OGD-treated N2a cells through miR-760-3p.

### CHAC1 overexpression reversed the effects of ADSC-Exo on ferroptosis inhibition

**Fig. 8:**
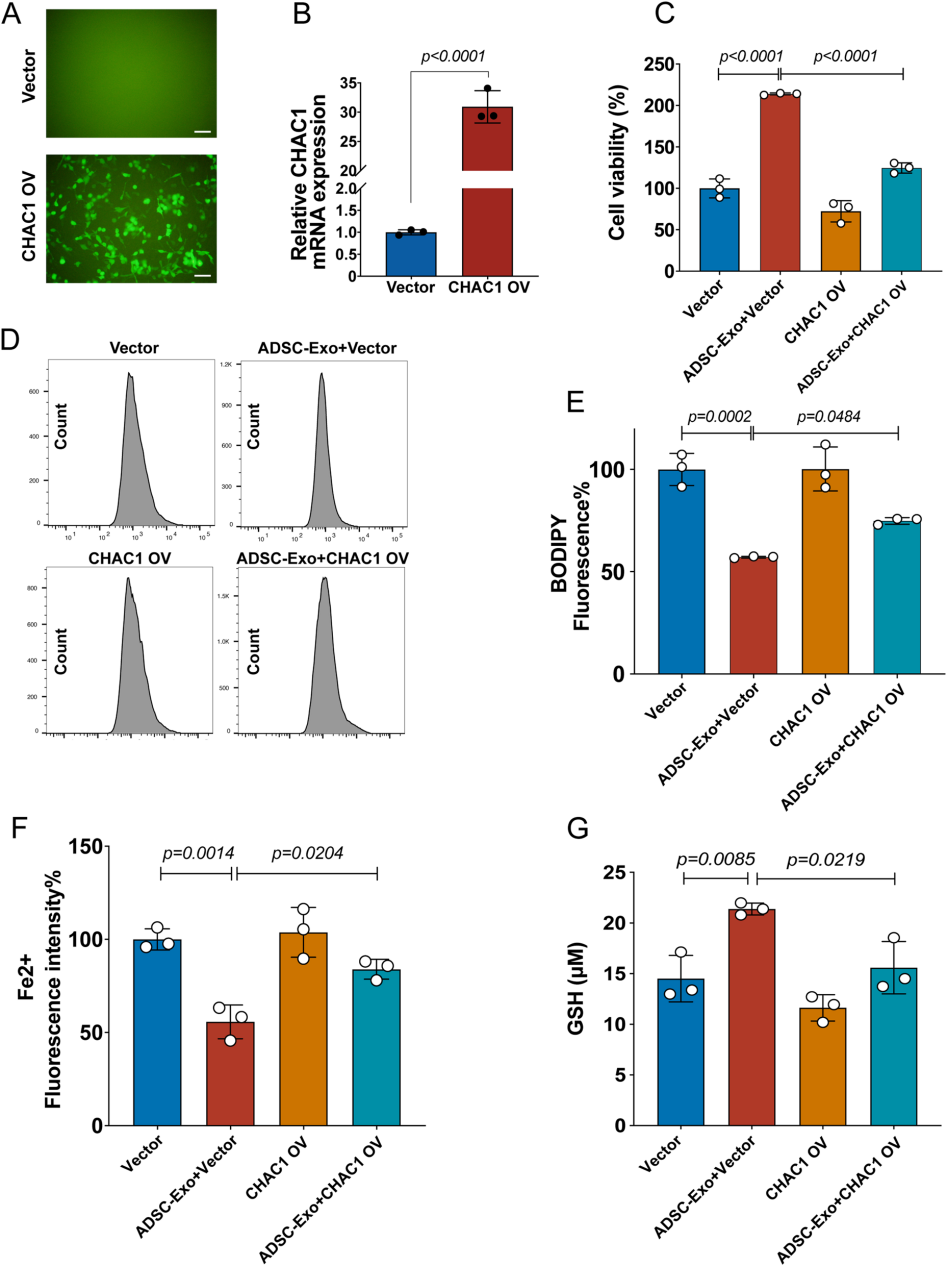
CHAC1 overexpression reversed the effects of ADSC-Exo on ferroptosis inhibition. **A** Transfection efficiency of the lentivirus virus was detected by fluorescence microscopy, Scale bars = 50 μm. **B** Overexpression of CHAC1 in N2a cells confirmed by RT-qPCR, *n* = 3. **C** The survival rate of OGD-treated N2a cells in different groups was assessed by CCK-8 assay, *n* = 3. **D**, **E** The lipid peroxidation of OGD-treated N2a cells in different groups was assessed by the C11-BODIPY probe**,** *n* = 3. **F** The ferrous iron level (Fe^2+^) of OGD-treated N2a cells at different groups was assessed by the FerroOrange probe**,** *n* = 3. **G** The GSH level of OGD-treated N2a cells in different groups was assessed by Glutathione Assay Kit, *n* = 3

To investigate whether the effect of ADSC-Exo on ferroptosis inhibition was associated with CHAC1, we assessed the ferroptosis-related outcomes in OGD-treated N2a cells. Transfection efficiency of the lentivirus virus was detected by fluorescence microscopy (Fig. 8A), and RT-qPCR confirmed the overexpression of CHAC1 in N2a cells (Fig. 8B). The CCK-8 assay, C11-BODIPY probe, FerroOrange probe, and GSH assay showed that ADSC-Exo increased the survival rate (Fig. 8C) and the GSH levels (Fig. 8G), and reduced the lipid peroxidation levels (Fig. 8D–E) and the ferrous iron levels (Fig. 8F) of OGD-treated N2a cells, while these effects of ADSC-Exo were reversed by CHAC1 overexpression, indicating that ADSC-Exo inhibit ferroptosis in OGD-treated N2a cells through down-regulating the expression of CHAC1. All these results suggested that miR-760-3p derived from ADSC-Exo can inhibit ferroptosis through down-regulating the CHAC1 expression in OGD-treated N2a cells.

## Discussion

Ferroptosis is a recently discovered form of cell death playing an irreplaceable role in ischemic stroke [12]. Recent studies have suggested that some ferroptosis-related genes can be served as diagnostic biomarkers for ischemic stroke, providing some evidence about the vital role of ferroptosis in ischemic stroke [31, 32]. Additionally, from a biochemical standpoint, brain tissue is rich in phospholipids, and lipid peroxidation is an important feature of brain injury. During ischemic stroke, excessive and lethal accumulation of lipid reactive oxygen species (ROS) in biofilms leads to glutathione depletion and inactivation of GPX4 [33]. Excessive glutamate released by ischemic neurons can inhibit the cystine/glutamate antiporter (system XC-) contributing to glutamate-induced neurotoxicity [34]. Iron ion accumulation and iron-dependent lipid peroxidation are also increased during the pathological process of ischemic stroke [35]. The structural and functional damage of the blood–brain barrier also facilitates the entry of iron from the blood into the brain parenchyma, ultimately triggering neuronal ferroptosis [8, 36]. Furthermore, previous studies have shown that ischemia-induced ferroptosis manifests as increased lipid peroxidation with decreased GSH levels. Inhibition of ACSL4 expression reduces lipid peroxidation and attenuates brain damage after stroke [8]. More importantly, the ferroptosis inhibitors Liproxstatin-1 and Ferrostatin-1 can significantly reduce infarct volume and neurological deficits, attenuating cerebral ischemia–reperfusion injury in mice[11, 12]. Therefore, these findings confirm the close relationship between ferroptosis and ischemic stroke. After ischemic stroke, ferroptosis has closely related to other pathological processes such as inflammation [37] and oxidative stress [38] in ischemic stroke. It may be necessary to dialectically integrate, rather than separate, ferroptosis from other relevant pathological processes in order to find better therapeutic targets for stroke. To date, the detailed molecular mechanism of ferroptosis and measures to deal with it in ischemic stroke are still largely unclear. In this study, we identified that ADSC-Exo contain abundant miR-760-3p that can regulate the expression of the ferroptosis-related gene CHAC1. We isolated ADSC-Exo and confirmed its characteristics. Then, we identified that IN administration of ADSC-Exo at the acute phase can reduce the expression of ferroptosis-related proteins inhibiting ferroptosis, and promote the recovery of neurological function in ischemic stroke mice. We also confirmed that IN administration of ADSC-Exo achieve these effects at least partly by delivering miR-760-3p to neurons, which down-regulated the expression of CHAC1, inhibiting the ferroptosis of neurons.

Previous studies have revealed that ferroptosis was induced in ischemic stroke as evidenced by the increased ferroptosis-related lipid peroxidation products levels and reduced antioxidants levels like GSH, and administration of ferroptosis inhibitors can improve I/R injury [39]. In this study, obvious high expression of ferroptosis-promoted protein and low expression of ferroptosis-inhibited protein were detected, and the ferroptosis progressed to the most serious stage at 3d after I/R. We found that ACSL4 was suppressed instead of increased at 1d after I/R, which was consistent with that reported by a previous study, and this was explained as the suppression effect of HIF-1α in the early phase [10]. In combination with previous data, our findings strongly confirmed that ferroptosis indeed play a major role in ischemic stroke.

In order to explore the molecular mechanism and then identified the potential therapeutic targets for ferroptosis in ischemic stroke, we identified a ferroptosis-related DEG, CHAC1, by analyzing a rare dataset from stroke patients’ brain tissues. The target miRNA of CHAC1, miR-760-3p, was also identified, and bioinformatics analysis revealed that miR-760-3p is abundant in ADSC-Exo. Thus, we speculated that miR-760-3p/CHAC1 axis exerts vital roles in inhibiting ferroptosis with ADSC-Exo in ischemic stroke, and verified the expression of these two genes in different datasets and tissues from different species. CHAC1, ChaC Glutathione Specific Gamma-Glutamylcyclotransferase 1, is a protein-coding gene. CHAC1 was reported to be a ferroptosis-related gene, and mainly explored in various cancers [40–42], while no related finding was reported in stroke disease. The research focus on miR-760-3p was rare, and only one study reported that it was related to inflammatory [43]. As miR-760-3p is abundant in ADSC-Exo, we believe that IN administration of ADSC-Exo can deliver miR-760-3p to target cells suffering from ferroptosis.

We successfully collected ADSC-Exo from ADSCs supernatant by ultracentrifugation. The characteristics of ADSC-Exo were confirmed by transmission electron microscopy, particle size analyzer, and Western blot. Additionally, the result of immunofluorescence images showed that the PKH26-labeled ADSC-Exo can be observed in the brain, which demonstrated that the delivery efficiency of IN administration in delivering ADSC-Exo to the brain is high. Mesenchymal stem cells (MSCs), a heterogeneous subset of stromal cells, can be easily isolated from adipose, bone marrow, and muscle tissue [21]. Previous studies confirmed the efficacy of MSCs in reducing neurological deficits after ischemic stroke in preclinical and clinical research [44, 45]. Much attention has been focused on adipose-derived mesenchymal stem cells (ADSCs), as they have the advantages of being abundant and easy to obtain [46]. Various studies indicated that ADSCs can alleviate the extent of damage in ischemic stroke by promoting angiogenesis and synaptic remodeling, and reducing apoptosis, inflammatory factors levels, and glial scar formation [47–50] However, it is hard for ADSCs to survive after transplantation, it is speculated that ADSCs achieve these therapeutic effects mainly through paracrine pathways, and ADSC-Exo were regard as the substitute of ADSCs [49]. The therapeutic effects of ADSC-Exo have been explored and confirmed in hemorrhage stroke, and the ferroptosis-inhibition effect of them was also identified [21, 22]. Lin et al. showed that ADSC-Exo can inhibit ferroptosis in the model of acute liver injury by maintaining the stability of SLC7A11 [21]. This study does not specifically explore which content of ADSC-Exo plays an anti-ferroptosis role, but it shows that ADSC-Exo has an important protective effect on SLC7A11, which is able to transport cystine into cells to play an antioxidant role through glutathione. In the present study, CHAC1 also promotes ferroptosis by degrading glutathione. Therefore, this study provides a certain basis for our research. Xia et al. showed that ADSC-Exo inhibits ferroptosis by targeting IRP2 in intracerebral hemorrhage mice through miR-19b-3p [22]. This study demonstrated that ADSC-Exo contain miRNAs that can regulate the ferroptosis-related gene providing the basis for exploring miR-760-3p in the present study.

However, whether ADSC-Exo can inhibit ferroptosis in ischemic stroke was still unclear. In this study, we found that IN administration of ADSC-Exo at acute phase can regulate the ferroptosis-related markers at 3 d after I/R, and improve the neurological deficits, grasping function, coordination function, and motor function of ischemic stroke mice. These results indicated that IN administration of ADSC-Exo indeed inhibit ferroptosis in ischemic stroke mice improving their neurobehavioral function. As the neurobehavioral function is directly related to neurons [51], we speculated that ADSC-Exo inhibit the ferroptosis of neurons in ischemic stroke mice to a certain degree.

To confirm our previous speculation that whether ADSC-Exo inhibit ferroptosis of neurons, we conducted immunofluorescence staining in vivo. The results showed that the co-localization of NeuN, a neuron marker, with CHAC1 obviously, while no co-localization of CHAC1 with markers of other cells was identified. These results indicated that CHAC1 only up-regulated in neurons after I/R, and further confirmed that ADSC-Exo improve the recovery of neurological function through inhibiting ferroptosis of neurons. Subsequently, we further investigated these findings in vitro using the OGD model. We find that administration of ADSC-Exo can inhibit ferroptosis in OGD-treated N2a cells, while these therapeutic effects were partial erasure when added miR-760-3p inhibit or overexpressed CHAC1. These results further confirmed our previous findings in vivo*.* To our knowledge, this is the first time that IN administration of ADSC-Exo can inhibit ferroptosis via the miR-760-3p/ CHAC1 axis in ischemic stroke.

## Conclusion

Collectively, the present study successfully designed and prepared ADSC-Exo. We demonstrated the delivery efficiency of IN administration in delivering ADSC-Exo to the brain, investigated the efficacy of ADSC-Exo for ischemic stroke using a mouse MCAO model, and explored the molecular mechanism through which ADSC-Exo exert their function. Our findings indicated that ADSC-Exo can inhibit ferroptosis in vivo and in vitro. Additionally, we identified that ADSC-Exo can inhibit ferroptosis via delivering miR-760-3p to neurons down-regulating the expression of CHAC1. This study provided a potential therapeutic strategy for ischemic stroke.

## Data Availability

The datasets used and/or analyzed during the current study are available from the corresponding author upon reasonable request.
